# An eleven-gene risk model associated with lymph node metastasis predicts overall survival in lung adenocarcinoma

**DOI:** 10.1038/s41598-023-27544-0

**Published:** 2023-04-26

**Authors:** Yan Zhao, Wei Shi, Qiong Tang

**Affiliations:** Department of Respiratory, Tianjin Union Medical Center, Nankai University, Jieyuan Road 190, Hongqiao District, Tianjin, China

**Keywords:** Cell biology, Cell division, Cell growth

## Abstract

Lung adenocarcinoma (LUAD) occupies major causes of tumor death. Identifying potential prognostic risk genes is crucial to predict the overall survival of patients with LUAD. In this study, we constructed and proved an 11-gene risk signature. This prognostic signature divided LUAD patients into low- and high-risk groups. The model outperformed in prognostic accuracy at varying follow-up times (AUC for 3 years: 0.699, 5 years: 0.713, and 7 years: 0.716). Two GEO datasets also indicate the great accuracy of the risk signature (AUC = 782 and 771, respectively). Multivariate analysis identified 4 independent risk factors including stage N (HR 1.320, 95% CI 1.102–1.581, *P* = 0.003), stage T (HR 3.159, 95% CI 1.920–3.959, *P* < 0.001), tumor status (HR 5.688, 95% CI 3.883–8.334, *P* < 0.001), and the 11-gene risk model (HR 2.823, 95% CI 1.928–4.133, *P* < 0.001). The performance of the nomogram was good in the TCGA database (AUC = 0.806, 0.798, and 0.818 for 3-, 5- and 7-year survival). The subgroup analysis in different age, gender, tumor status, clinical stage, and recurrence stratifications indicated that the accuracy was high in different subgroups (all *P* < 0.05). Briefly, our work established an 11-gene risk model and a nomogram merging the model with clinicopathological characteristics to facilitate individual prediction of LUAD patients for clinicians.

## Introduction

Lung cancer is a leading cause of cancer-related death worldwide. Non-small cell lung cancer (NSCLC) consists of two common histological subtypes: lung adenocarcinoma (LUAD) and lung squamous cell carcinoma (LUSC), accounting for the majority of lung cancer cases^1^. LUAD is the main histological subtype of NSCLC, accounting for over 40% of lung cancer incidence rate^2^. For patients with LUAD, early surgical resection is the current standard treatment. After surgery, patients usually receive additional chemotherapy, and the survival rate can be improved by 5% to 10%^3^.

Despite recent improvements in adjuvant and neoadjuvant therapy, the 5-year survival rate of LUAD patients is still relatively low^4^. At present, the clinical evaluation of the prognosis of LUAD mainly depends on the TNM stage at the time of diagnosis, which cannot provide accurate individualized prognosis prediction. However, various disease outcomes have been found in patients with similar clinical and pathological features, suggesting that the clinical prognostic factors currently used may not be sufficient to consistently predict individual clinical outcomes^5^. Markers that can reliably evaluate prognosis would have significant value in guiding the treatment of LUAD^6^. This emphasizes the need to identify reliable prognostic markers with higher sensitivity and accuracy in LUAD. Therefore, combining the results of multiple studies is expected to obtain more reliable prognostic characteristics.

Currently, transcriptome profiling has widely been used to characterize prognostic signatures in patients with lung cancer, and has generated a number of candidate biomarkers with potential clinical values^7^. At the same time, machine learning methods have been introduced, adapted and applied to gene and genome data to clarify complex cellular mechanisms, identify molecular features, and predict clinical outcomes from large biomedical data sets^8,9^. Previous studies have highlighted various models that integrate clinical information and gene expression profiles in public databases, which may have the potential to predict the prognosis of LUAD. In recent years, more and more prognostic biomarkers of LUAD have been found by analyzing the clinical information and expression profile in the public database^10,11^. One study identified 2472 significant survival-related genes of LUAD patients by analyzing TCGA and GEO databases. Finally, 16 genes were found to be highly correlated with patients’ risk^12^. In another study, Wang et al. identified a four-gene signature that could effectively stratify a high-risk subset of LUAD with lymph node metastasis patients by analyzing the expression profiles of LUAD patients in TCGA database^13^. These studies show that using public database resources to develop predictive risk models has great potential. However, the prediction effect of the mentioned risk signature and adequate verification is not specific enough. Therefore, it is necessary to continue to explore the genetic and polygenic features related to the prognosis of LUAD to improve the predictive accuracy and external validation.

In this study, we sought to identify and validate robust and reliable prognostic features of predictions associated with overall survival (OS). In this study, we downloaded LUAD mRNA expression profiles from TCGA data sets and related geographic data sets. The LUAD prediction model based on 11 genes was successfully established by lasso regression and verified on the geographic data set. Our study provides a new method to help predict the prognosis of clinical LUAD patients, and provides more insights into the molecular mechanism of this common and devastating disease.

## Materials and methods

### Acquisition and procession of LUAD datasets

TCGA LUAD RNA sequence data and clinical data were downloaded from TCGA database (https://portal.gdc.cancer.gov) a large‐scale public data platform portal, measured experimentally using the Illumina HiSeq 2000 RNA Sequencing platform (October 13, 2017). According to the mRNA expression profile, the differentially expressed genes (DEGs) were screened by R-package "edge", and the normal samples were set as the control group (| logFC |> 1.0, adjusted *p* value < 0.05)^14^. In terms of geographic data, we chose two GEO datasets for they were LUAD patients and they had sufficient patients number. Therefore, GSE31210 (including 226 lung adenocarcinoma samples), GSE72094 (including 442 lung adenocarcinoma tissues), and corresponding clinical information are downloaded from the geographic database (https://www.ncbi.nlm.nih.gov/geo/). Our research excluded any samples that had missing or insufficient data on age, stage, recurrence (no or yes), tumor status (with tumor or tumor free), or living status (alive or dead), and survival duration. In the correlation analysis with the clinicopathological features of LUAD patients, samples such as "unknown", "TX" and "NX" were excluded.

### Bioinformatics analysis

The gene co-expression network between normal and cancer samples of TCGA LUAD data set was established by using weighted gene correlation network (WGCNA). Firstly, the variance stable conversion algorithm implemented in DEseq2 software package is used to normalize the count data^15^. Then, before network analysis, LUAD data is evaluated by clustering to check whether there are any obvious outliers. Then the optimal soft threshold is selected by WGCNA R package to maintain sufficient connectivity and make the gene network close to scale-free topology^16^. Parameter β is used to penalize weak correlations and emphasize strong correlations between genes. Furthermore, we evaluated the correlation between the lymph node metastasis (LNM) and the modules to identify the module that had the most significant correlation to LNM. Gene ontology analysis (GO) is extensively used to identify unique biological properties from high-throughput transcriptome or genome data, where gene functions are classified into biological process (BP), molecular function (MF), and cellular components (CC)^17^. The Kyoto Encyclopedia of Genes and Genomes (KEGG) is a collection of databases dealing with genomes, diseases, biological pathways, drugs and chemical materials^18^. To further explore the biological significance of DEGs, we performed a GO classification and a KEGG pathway analysis using the “clusterProfiler” package^19^.

### Construction and validation of the risk model

The key modules significantly related to LNM in WGCNA were selected for LASSO regression pipeline to narrow the range of target genes. Lasso regression analysis was used to reduce the collinearity between genes and prevent overfitting of prognostic risk model variables. This method is very popular in machine learning and is implemented by R package "*glmnet*"^20^. Genes selected from LASSO regression analysis were used to construct risk score characteristics. The signature based risk score is calculated by the following formula^21^:

$${\text{Risk score}} = \sum {\text{n}}_{{\text{i}}} = \sum \left( {{\text{Coefi*x}}_{{\text{i}}} } \right)$$

Coefi is the coefficient and x_i_ is the z-score transformed relative expression value of each selected gene. We calculate the score of each patient and categorize the whole population into high- and low-risk groups according to the median value of risk score, thus building a risk predictive model. A log-rank test compared the difference of OS between the two subgroups. Receiver operating characteristic (ROC) curves were drawn using the R package “survivalROC” for validation of the risk model and the AUC values of 3, 5 and 7-year survival were calculated^22^. To further evaluate whether the risk factor classifier was an independent risk factor for OS, we carried out univariate and multivariate Cox regression analyses with risk score and combined it with other clinical features to identify independent risk factors, and then construct a nomogram with these independent prognostic factors by “rms” package. Calibration curves were drawn and the concordance index (C-index) was computed to assess the accuracy of the nomogram^23^. The prognostic risk value of each patient was calculated by nomogram, and then the whole group was divided into three subgroups on average according to the total score of nomogram, including low, medium and high subgroups. Then the prognosis of nomogram was evaluated by tdROC analysis and survival rate estimation. The area under the tdROC curve (AUC) calculated by the "timeROC" software package indicates the accuracy of prediction or prognosis. The survival estimates of patients in the three subgroups were analyzed by the "survival" packet in R using the Kaplan Meier method. Two independent data sets GSE31210 and GSE72094 were used to confirm the prediction ability of the risk model. In addition, a *p* value < 0.05 was considered statistically significant. Moreover, immune scores of different subgroups were calculated with the package “estimate”, and correlations between risk scores and immune scores, stromal scores, and ESTIMATE score of each LUAD tumor sample were investigated.

### Statistical analysis

SPSS version (v. 21.0) and GraphPad Prism (v. 8.0) were used for statistical analysis and generating figures. Paired t-test, unpaired t-test and one-way ANOVA were used to compare the gene expression of different groups. Chi-square test was used to analyze the correlation between gene expression and clinicopathological factors. Kaplan Meier survival analysis of TCGA and geo database was used to study the prognostic significance of risk model and calculate log rank p value. Cox proportional hazards regression model was used for univariate and multivariate analysis of survival. Prognostic factors in univariate analysis were included in subsequent multivariate analysis. *P* < 0.05 was considered statistically significant.

## Results

### Identification of DEGs and WGCNA analysis

**Figure 1:**
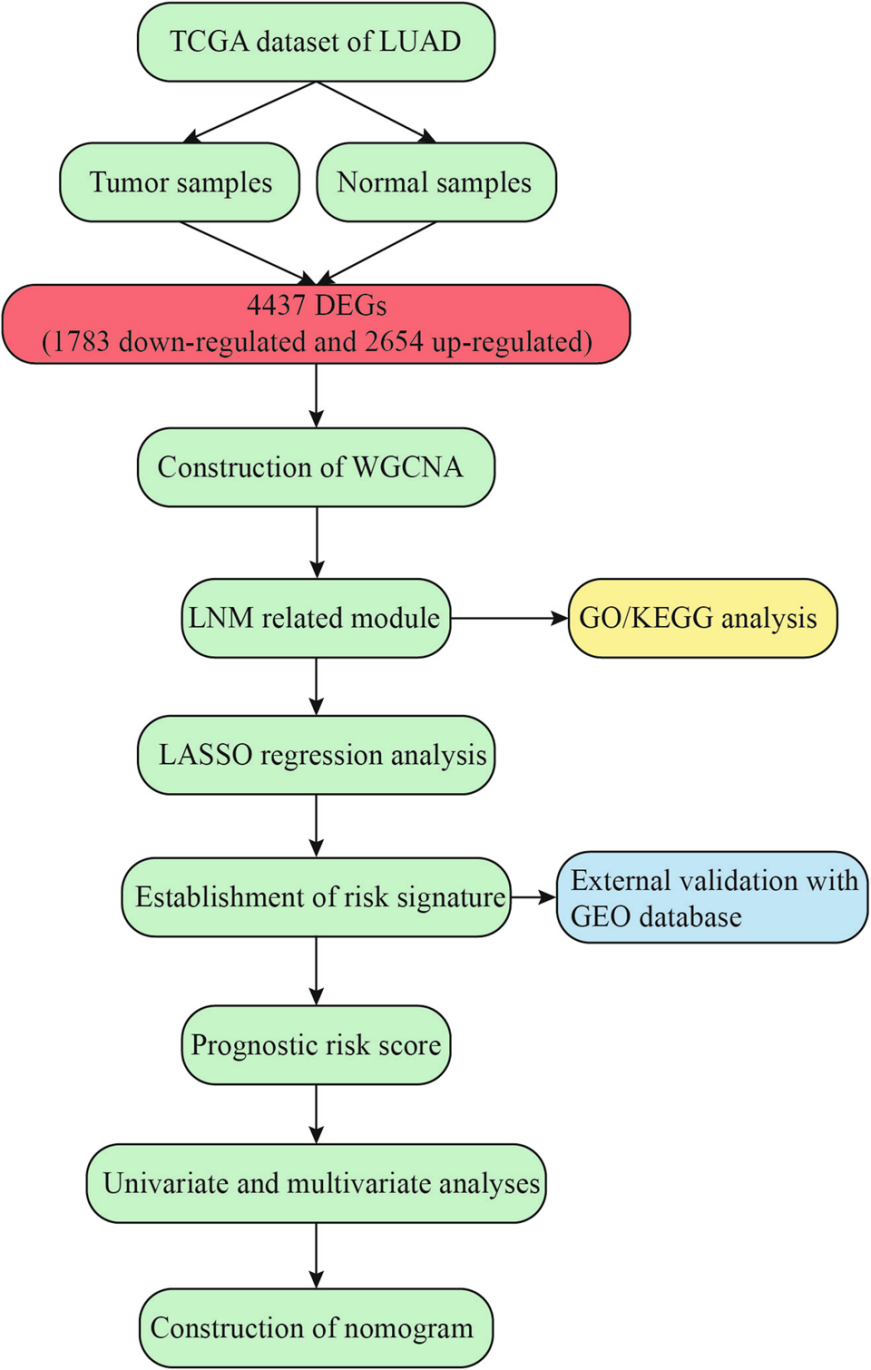
The flow chart of the study design and analysis. *TCGA* The Cancer Genome Atlas, *LUAD* lung adenocarcinoma, *DEGs* differentially expressed genes, *WGCNA* Weighted Gene Correlation Network Analysis, *LNM* lymph node metastasis, *GO* gene ontology, *KEGG* Kyoto Encyclopedia of Genes and Genomes, *LASSO* least absolute shrinkage and selection operator, *GEO* Gene Expression Omnibus database.

**Figure 2:**
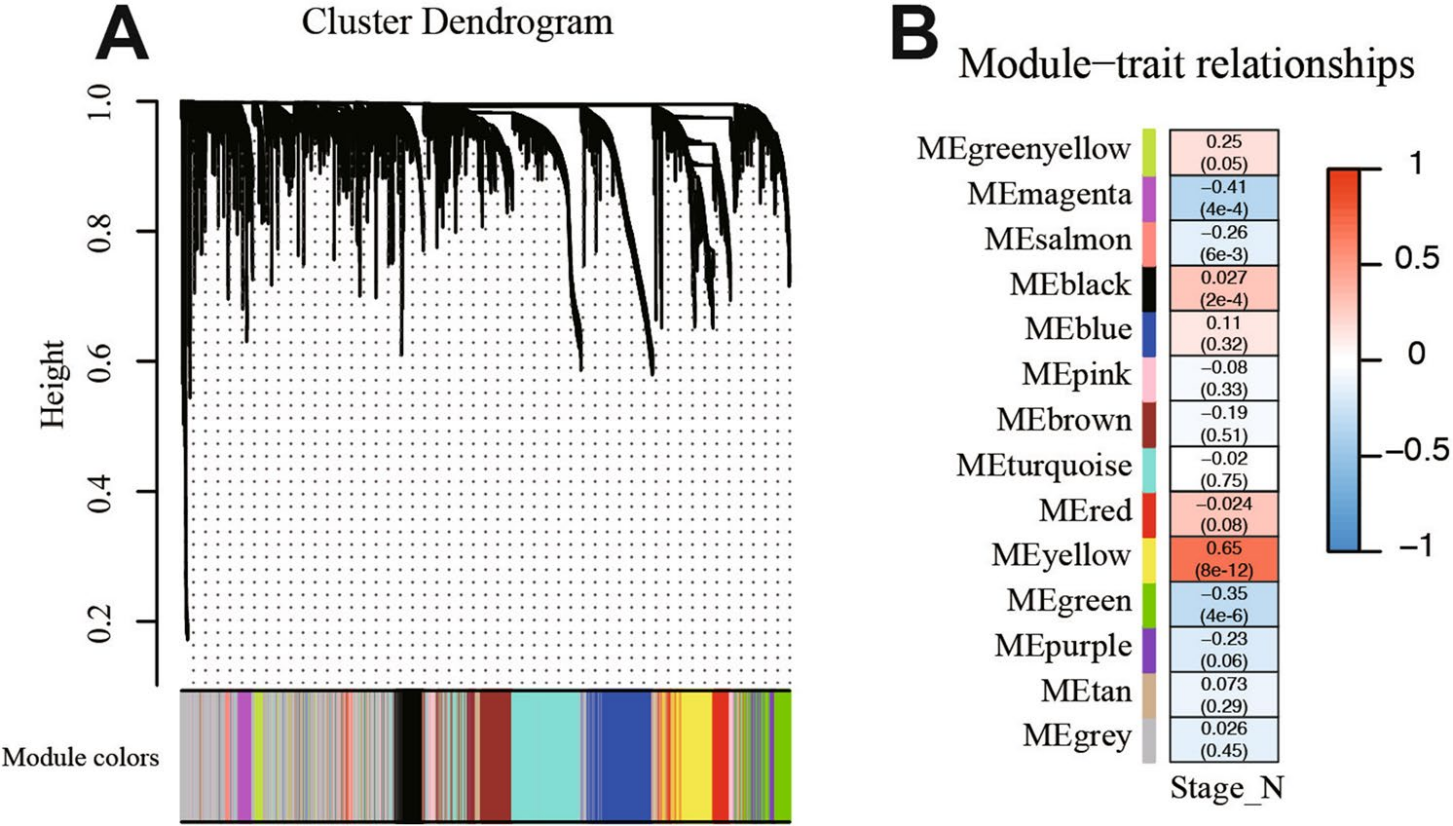
Identification of DEGs and WGCNA construction. (**A**) Clustering dendrogram of genes in LUAD tissues. (**B**) Correlation between modules and risk model.

In general, we extracted 517 LUAD patients with clinical and pathological diagnosis from TCGA database and analyzed these data, as shown in the flow chart (Fig. 1). The mRNA expression profile and clinical data of LUAD were downloaded from TCGA database. Finally, 4437 DEGs were obtained by differential analysis using R-package, including 2654 up-regulated genes and 1783 down-regulated genes. In order to better understand the relationship between LNM and molecular groups, we extracted RNA sequence data and performed WGCNA. One of the most critical parameters is the power value, which mainly affects the independence and average connectivity of the co-expression module. Using WGCNA software package, the co-expression network of DEGs was analyzed and selected β = 5. The power of 5 ensures a scale-free network (Fig. S1A). At the same time, the fitting degree of scale-free topology model is 0.98. Therefore, the network conforms to the power-law distribution and is closer to the real biological network state (Fig. S1B). These co-expression modules were then constructed and divided into 14 meaningful modules (Fig. 2A). By analyzing the association between gene module and LNM, we found that the Yellow module had the highest correlation with N stage (LNM) (COR = 0.65, *P* = 8e−12) (Fig. 2B). There were 134 genes in the Yellow module, which were further used for subsequent analysis.

### GO/KEGG enrichment analysis of key modulus

**Figure 3:**
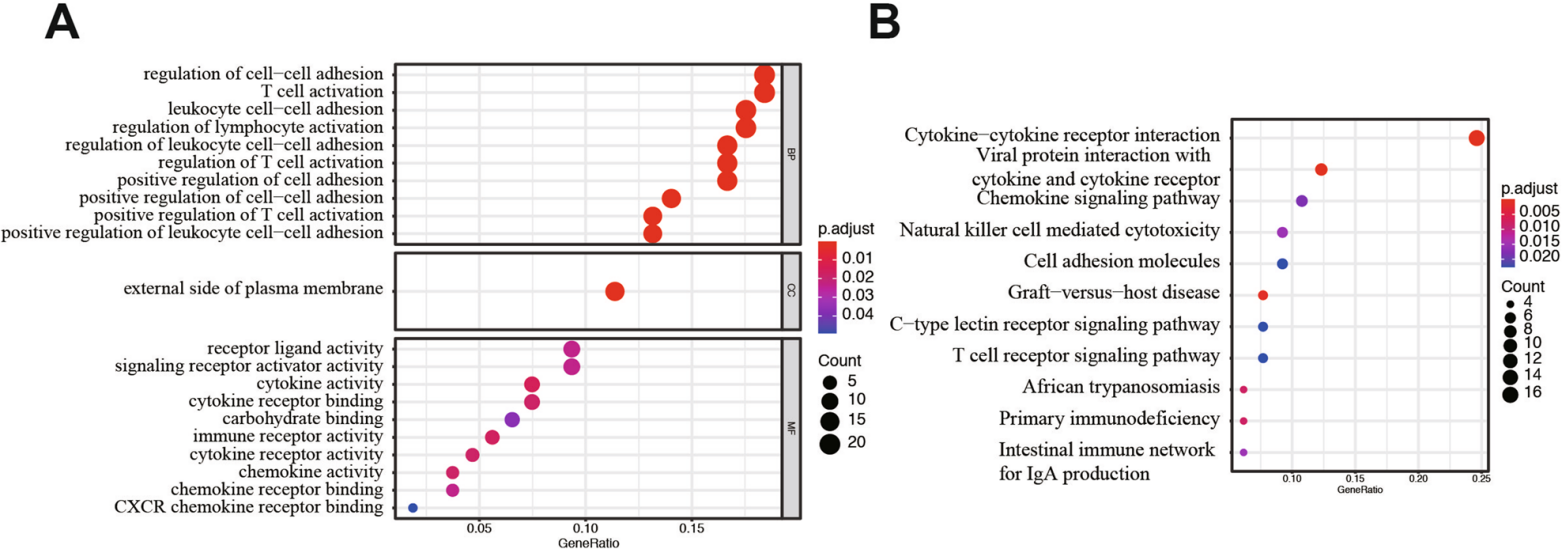
GO and KEGG analysis of DEGs from TCGA LUAD database. (**A**) GO analysis. (**B**) KEGG analysis.

To elucidate the potential function of these 134 genes, GO and KEGG analyses were carried out. As shown in Fig. 3A, the GO plot significant terms were various and some of them were as followed: “regulation of cell–cell adhesion”, “T cell activation”, and “positive regulation of leukocyte cell–cell adhesion”. In addition, KEGG analysis revealed that these 134 DEGs were significantly enriched in pathways in “Cytokine–cytokine receptor interaction”, “Viral protein interaction with cytokine and cytokine receptor”, “Chemokine signaling pathway”, “Cell adhesion molecules”, and “T cell receptor signaling pathway” (Fig. 3B). These results indicated that these DEGs were distributed in cell adhesion and immune cell related pathway, which have been proved to play a pivotal role in the tumorigenesis and progression of LUAD. What’ more, these functions also implied us about the mechanism of progression for LUAD patients.

### Construction and validation of risk model

**Figure 4:**
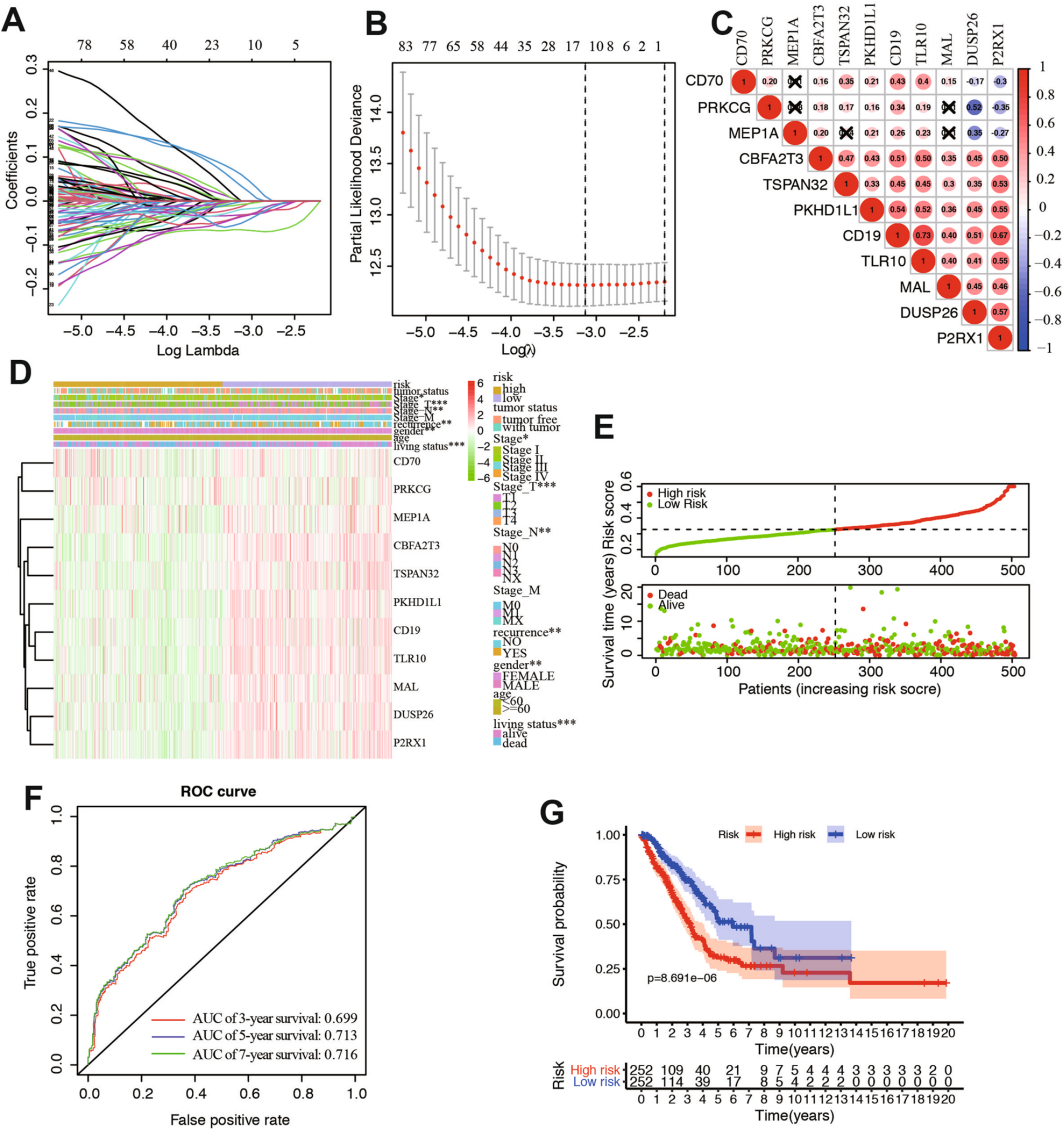
Identification of prognostic genes in LUAD patients. (**A**,**B**) LASSO regression model. (**C**) Spearman correlation analysis of the 11 genes expression. (**D**) Heatmap and clinicopathological features of the two groups, Chi-square test was used for correlation between clinical and cluster, *P < 0.05, **P < 0.01, and ***P < 0.001. (**E**) Risk score distribution in the two groups. (**F**) Kaplan–Meier survival analysis of the low and high-risk group. (**G**) Time-dependent ROC curve for 3-, 5-, and 7-year survival prediction.

In order to develop a risk classifier related to LNM to predict the prognosis of LUAD, the expression profile data of 134 genes in the Yellow module were used for lasso regression model, and the penalty parameter lambda was selected by cross validation method to obtain relatively independent characteristic genes for subsequent model analysis (Fig. 4A,B).Finally, eleven genes were found with regression coefficients including CD70, PRKCG, MEP1A, CBFA2T3, TSPAN32, PKHD1L1, CD19, TLR10, MAL, DUSP26, and P2RX1 (Table 1). The risk score formulation for this signature were established as following: risk signature = CD70 * 0.06048 + PRKCG * 0.03435 − MEP1A * 0.00103 − CBFA2T3 * 0.06531 − TSPAN32 * 0.05082 − PKHD1L1 * 0.01894 − CD19 * 0.01312 − TLR10 * 0.03594 – MAL * 0.03171 − DUSP26 * 0.00774 − P2RX1 * 0.03343. The expression of the eleven genes were significantly correlated with each other especially between CD19 and P2RX1, TLR10 and P2RX1, PKHD1L1 and P2RX1 in the dataset (Fig. 4C). The expression heatmap of the 11 genes in the high and low risk groups was plotted and the clinicopathologic differences between the two groups were shown in the heatmap as well. The results concluded that with the increase of the risk score, the expression levels of CD70 and PRKCG gradually increased, while the expression levels of MEP1A, CBFA2T3, TSPAN32, PKHD1L1, CD19, TLR10, MAL, DUSP26, and P2RX1 gradually decreased. Moreover, there were significant differences between the high-risk group and the low-risk group in different clinicopathological features such as stage, recurrence, living status, and gender (Fig. 4D). The risk score of each sample was calculated according to the 11 independent prognostic characteristic genes. Therefore, LUAD patients with follow-up information were divided into two groups: low-risk group (n = 252) and high-risk group (n = 252) (Fig. 4E). ROC curves were drawn to verify the risk assessment model, and the AUC values of 3, 5 and 7-year survival were 0.699, 0.713 and 0.716, respectively (Fig. 4F). Then, the Kaplan–Meier curve and log-rank test suggested that patients in the high-risk group have significantly worse overall survival compared to those in the low-risk group (*P*-value = 8.69e−06) (Fig. 4G). We compared the presence or absence of driver mutations including ROS1, BRAF, EGFR, ALK, and HER2 are closely related to therapeutic efficacy and prognosis in LUAD. Therefore, we compared the proportion of mutation for these genes and the results showed that different oncogenic driver mutations were different in high and low risk groups (Fig. S2). The results showed that the risk score decreased with the increase of stromal score (Fig. S3A), immune score (Fig. S3B), and ESTIMATE score (Fig. S3C). The above results suggested that the 11-gene signature-based risk assessment model had certain predictive value for the prognosis. In addition, the accuracy of this model and its correlation with prognosis are higher than previous similar studies.

**Table 1:** Genes from LASSO regression and correlated coefficient value.

Gene names	Coefficient
CD70	0.06048
PRKCG	0.03435
MEP1A	− 0.00103
CBFA2T3	− 0.06531
TSPAN32	− 0.05082
PKHD1L1	− 0.01894
CD19	− 0.01312
TLR10	− 0.03594
MAL	− 0.03171
DUSP26	− 0.00774
P2RX1	− 0.03343
Risk score	Low: < 0.32796
	High: ≥ 0.32796

*LASSO* least absolute shrinkage and selection operator.

### Development and evaluation of a nomogram for OS prediction in LUAD

**Figure 5:**
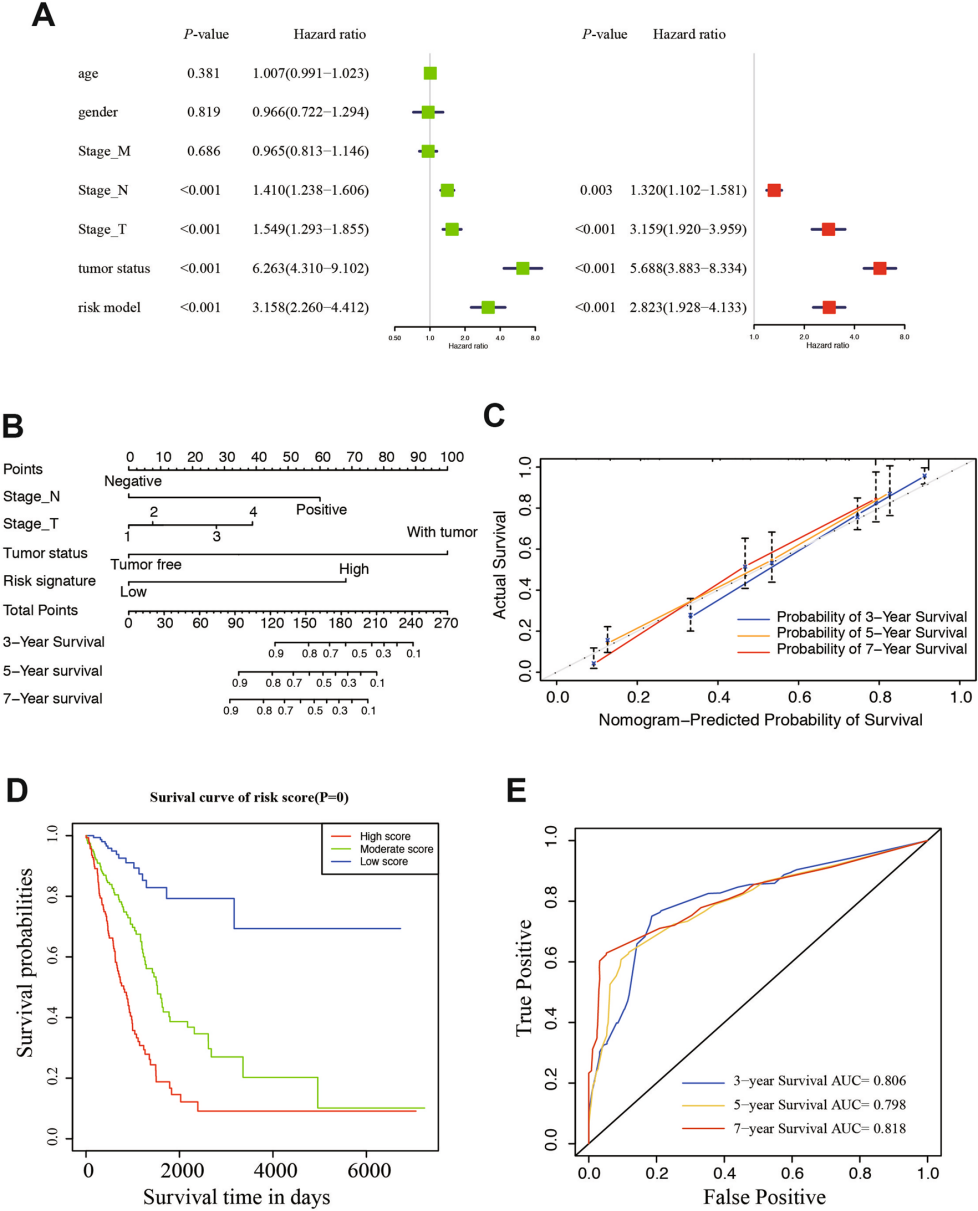
Establishment of a nomogram for survival prediction of LUAD patients. (**A**) Univariate and multivariate analyses of the association between clinicopathological factors and overall survival of LUAD patients. Hazard Ratios (HR), 95% CI, 95% confidence intervals. (**B**) Nomogram including the 11-gene risk signature and clinicopathological features. (**C**) Calibration plot of the nomogram-predicted probability and actual survival in training and validation cohorts. (**D**) Kaplan–Meier survival analysis of the 3 subgroups. (**E**) Time dependent ROC curves for the nomogram predicting 3-, 5-, 7-year survival.

To develop a quantitative method that could predict the probability of overall survival, we used a nomogram to build a predictive model. First, univariate and multivariate analyses were conducted to acquire the independent risk factors for overall survival. In multivariate analysis, NSCLC patients with advanced stage N (HR 1.320, 95% CI 1.102–1.581, *P* = 0.003), stage T (HR 3.159, 95% CI 1.920–3.959, *P* < 0.001), tumor status (HR 5.688, 95% CI 3.883–8.334, *P* < 0.001), and risk model (HR 2.823, 95% CI 1.928–4.133, *P* < 0.001) were associated with prognosis (Fig. 5A). The nomogram was generated to predict the prognosis on the basis of the multivariate analysis (*P* < 0.05) of OS in the TCGA LUAD patients (Fig. 5B). In the nomogram, tumor status contributed the most to the prognosis, followed by the risk model, stage_N, and stage_T. Discrimination and calibration of the nomogram were examined. The C-indexes of the nomogram was 0.768 (95% CI 0.712–0.805). Calibration plots showed an outstanding consistency and an acceptable fluctuation between predictions and actual observations for 3-/5-/7-year OS in the cohort (Fig. 5C). Then the TCGA cohort was evenly divided into three subgroups according to total score of the nomogram, high-score, moderate-score, and low-score groups (Table 2). LUAD patients in high-score group had significantly worse prognosis than those in moderate- and low-score groups (*P* < 0.001; Fig. 5D) for OS. We further test the three subgroups in in predicting relapse-free survival (RFS, Fig. S4A) and cancer-specific survival (CSS, Fig. S4B), both survival curve showed significant among the three risk groups. To further explore the efficiency of the nomogram, we conducted the ROC curve analysis and calculated the area under the ROC curve of the probability of overall survival. The results indicated that the AUCs of 3-/5-/7-year survival were 0.806, 0.798, and 0.818, respectively (Fig. 5E). Taken together, these results suggested that the nomogram based on the 11-gene risk model and clinical factors had significantly predictive function for the prognosis of LUAD patients. We have further improved the accuracy of prognostic prediction on the basis of previous studies.

**Table 2:** Corresponding risk score for each variable and total score.

Variables	Score
**Stage N**	
Negative	0
Positive	60
**Stage T**	
1	0
2	7.5
3	27.5
4	40
**Tumor status**	
Negative	0
Positive	100
**Risk signature**	
Low	0
High	70
**Risk score**	
Low risk	0–40
Moderate risk	40–107.5
High risk	> 107.5

### GEO validation of the risk model

**Figure 6:**
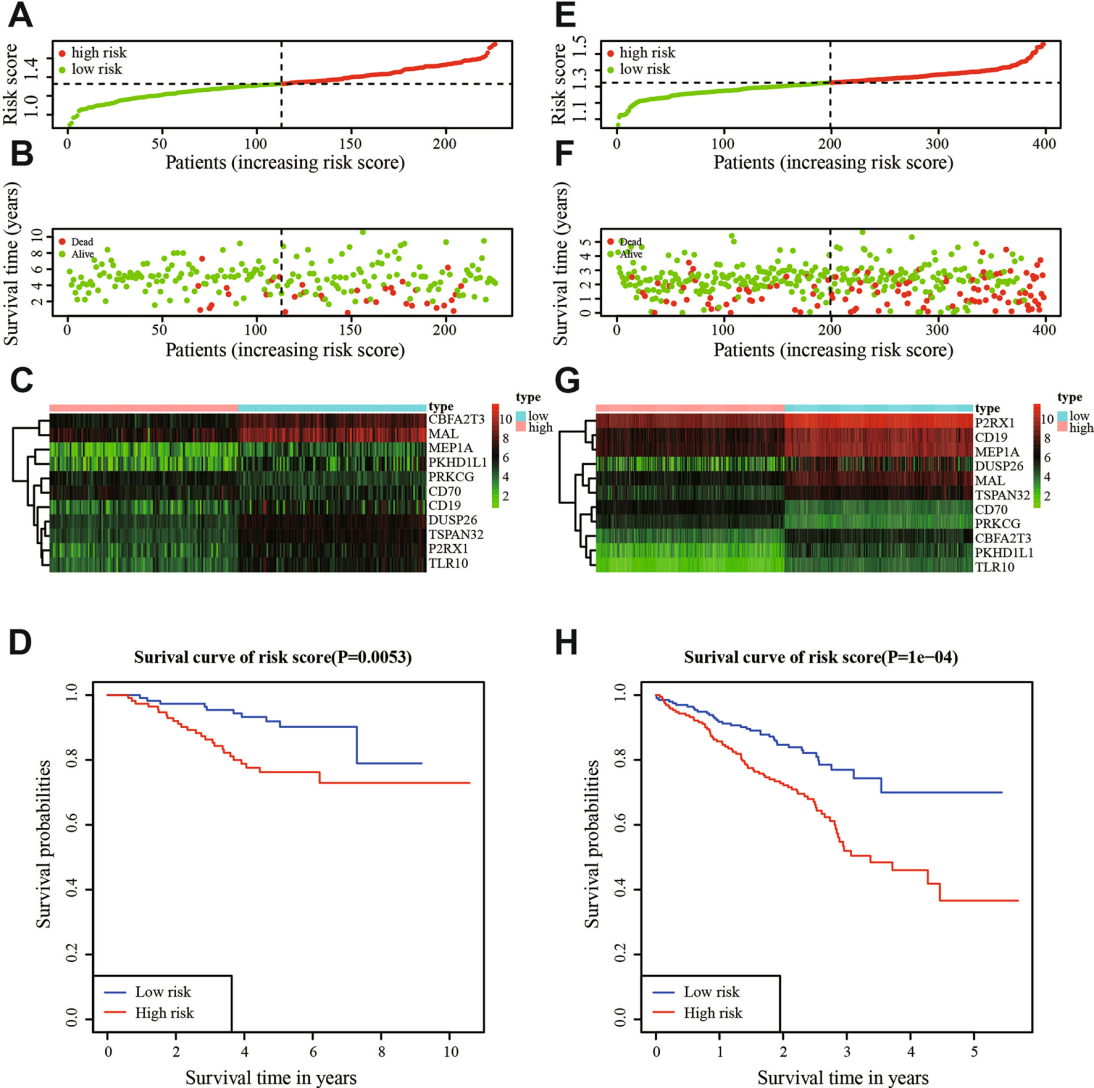
The distribution of risk scores, patients’ survival status, the heatmap of gene expression profiles, and ROC curves in the GEO cohort. (**A**) Distribution of risk scores in GSE31210. (**B**) Survival status of patients in GSE31210. (**C**) The heatmap of gene expression profiles in GSE31210. (**D**) Area under the ROC curve in GSE31210. (**E**) Distribution of risk scores in GSE72094. (**F**) Survival status of patients in GSE72094. (**G**) The heatmap of gene expression profiles in GSE72094. (**H**) Area under the ROC curve in GSE72094.

To further substantiate the availability and stability of this 11-gene risk model, we did the same analyses on the two external sets in GEO database (GSE31210 and GSE72094). For the external testing set GSE31210 (n = 226), the optimal cut-off value for classifying LUAD patients into high- and low-risk group was 1.32, with which the model successfully categorized 113 patients into the high-risk group and 113 patients into the low-risk group. The distribution of the risk score (Fig. 6A), survival status (Fig. 6B), and expression patterns of the 11-gene classifier (Fig. 6C) in two external sets also showed consistent results with the TCGA LUAD cohort. Higher risk score patients had poorer survival than lower risk score patients, and the former tended to have over-expression of CD70, PRKCG and lower expression of MEP1A, CBFA2T3, TSPAN32, PKHD1L1, CD19, TLR10, MAL, DUSP26,and P2RX1. The survival curve would distinguish the two different risk groups. (Fig. 6D). Likewise, validation on the external validation set GSE72094 (n = 442) showed consistent result that high-risk group patients (n = 221) had poorer OS compared with low-risk group patients (n = 221). And the risk model also differentiated the cohort into two risk groups (Fig. 6E,F). Gene expression in the two risk groups was significantly different (Fig. 6G). The survival curve suggested that the prognosis of patients in the high-risk group have significantly worse outcome compared with those in the low-risk group (*P*-value = 1.0e−04, Fig. 6H). These verifications show that the 11-gene risk model also has high accuracy in the external datasets.

### Prognostic value of the nomogram in different clinicopathological subgroups

**Figure 7:**
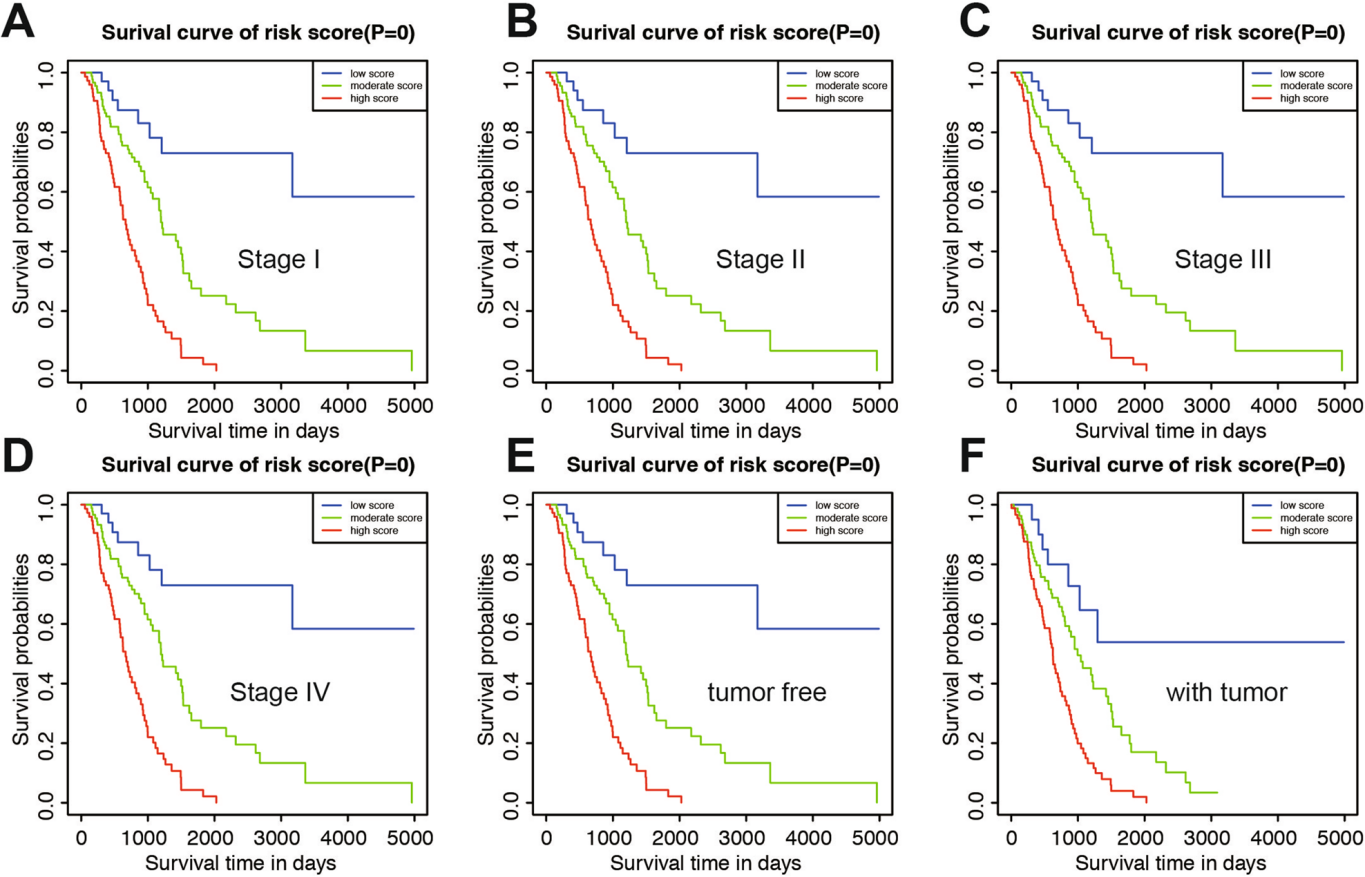
Prognostic value of the nomogram in different clinicopathological subgroups. (**A**) Stage I. (**B**) Stage II. (**C**) Stage III. (**D**) Stage IV. (**E**) Tumor free group. (**F**) With tumor group.

To further evaluate and test the survival assessment model, we stratified LUAD patients by different clinical characteristics in TCGA cohort according to stratification of the nomogram (Table 2). Results showed that, in all subgroups, including: clinical stage subgroups: stage I to stage IV, recurrence subgroups: yes or no; age subgroups: age < 60 and age ≥ 60; gender subgroups: male and female; tumor status subgroups: tumor free and with tumor, in the whole patients. Patients in the high-score groups had a shorter OS time than moderate- and low-score patients. The results showed that the predictive capability of the survival assessment model was effective in all of the clinicopathological subgroups for LUAD patients (Fig. 7A–F, Fig. S5A–F). Thus, the model had a certain reliability and practicability in evaluating prognosis of not only the whole set of patients, but each clinicopathological characteristics. This shows that the model has certain stability, which can select patients with poor prognosis from patients with good prognosis.

## Discussion

Accumulating evidence shows that lung adenocarcinoma patients with lymph node metastasis always exhibit poor responses to standard treatments and thus tend to have poor clinical outcomes^24–26^. Although great progress has been made in diagnosis and treatment, the prognosis of LUAD patients with lymph node metastasis is still poor. The established clinical survival indicators are mainly based on patients and cancer-related factors, such as TNM stage and grade, but the accuracy and specificity are also limited. A large number of studies have shown that the disorder of gene expression may be related to the occurrence, development and prognosis of tumors. Some genes are considered as prospective biomarkers to predict the prognosis of patients with NSCLC. For example, Zou et al. Proposed robust and reliable six gene features, which is of great significance in predicting DFS and OS in patients with NSCLC^27^. But few studies concentrated on LUAD patients. Therefore, our study aimed to identify novel molecular signatures integrated with established clinicopathological features to predict overall survival in LUAD patients.

In other studies that concentrating on LNM related signature, they generally compare LUAD patients with LNM and those without LNM^28^. In our study, we used WGCNA to find the correlation between gene modulus and significant clinical features of LNM. We first screened the DEGs, and then conducted WGCNA analysis, so as to avoid damaging the connectivity and correlation between genes in the gene module from WGCNA analysis. This is different from another LNM related study in lung cancer^13^. What’s more, the AUC of our nomogram is much higher than that in that study. Our model have been validated in two external models, and all these external validations have relative high accuracy. Finally, the stratified survival analysis proved to be high effective. We found 14 modules based on DEGs of LUAD. The correlation analysis shows that the correlation between yellow module and LNM is the best. GO and KEGG analysis showed that the function of yellow module was rich in "intercellular adhesion" and "chemokine signaling pathway". Early detection of adhesion molecules and signal regulation of lung cancer metastasis^29^. G protein-coupled receptors (GPCRs) played important roles in cell adhesion to the extracellular matrix and cell–cell communication, processes that are dysregulated in lung cancer cells^30^. Chemokine and its receptors also promote the migration and invasion of lung cancer cells^31^. The mechanism might regulated by the interaction between CXCR4 and EGFR and downstream PI3K/AKT pathway^32^.

Since LNM always affects tumor prognosis, we then performed lasso Cox regression analysis to identify key genes from hub module genes. Finally, 11 gene features were established from the hub module genes in the TCGA-LUAD cohort. Risk characteristics can also be used to divide LUAD patients into low-risk group and high-risk group to predict the overall survival for LUAD patients. As we can see, this model is not only predictive for OS, but stage_T and stage_M are differentially distributed in two risk groups as well. This is because patients with poor prognosis usually have large volume of tumors and are also prone to metastasis distant. There are usually significant differences in OS between the two groups, and the AUC of 7-year survival rate is as high as 0.716. In addition, the prognostic value of 11 gene markers was verified in two geographic data sets, indicating that the marker has stability and strong differentiation ability in dividing LUAD patients into high-risk and low-risk subgroups. Our results show that this risk feature can successfully identify high-risk and low-risk LUAD patients with significant differences in OS, and perform well in their survival prediction. To assess the independence of the 11-gene signature in predicting OS, we performed univariate and multivariate Cox regression analyses. After adjusting the effects of age, grade, pathological tumor stage in the regression analysis, the risk model of patients based on the 11-gene signature maintained a good correlation with OS. AUCs of the overall survival in the external GEO dataset reached 0.782 and 0.771, suggesting relatively ideal predictive accuracy. Overall, these results confirmed the prognostic power of the 11-gene model for predicting the OS of LUAD patients, and it was independent of other clinical features. Therefore, our predictive signature may help identify high-risk LUAD patients and make appropriate clinical follow-up plans accordingly. There were other studies constructing the risk signature with similar approaches, such as seven-gene prognostic signature, 14-gene signature, metabolism-related model in LUAD^33–36^, and the stem-cell-related signature in NSCLC^37^, our study focus on the lymph node metastasis with WGCNA method to recognize a gene modulus. The risk model and nomogram in our study was comprehensive and had a high predictive accuracy than other studies. In LUAD, histological subtypes according to the IASLC/ATS/ERS classification have also been reported to be a useful classification system reflecting patient prognosis. This classification is intended to support clinical practice, and research investigation and clinical trials. The IASLC/ATS/ERS classification has implications for strategic management of tissue, particularly for small biopsies and cytology samples, to maximize high-quality tissue available for molecular studies^38^. What’s more, existing studies have reported that most of these 11 key genes, including CD70, PRKCG, MEP1A, CBFA2T3, TSPAN32, PKHD1L1, CD19, TLR10, MAL, DUSP26, and P2RX1, are closely related to the development of multiple cancers. CD70 has different roles in predicting the prognosis of different cancers, and participate cancer progression through immunotherapy. Inhibition of CD70 function can be triggered by tumor-derived inhibitory cytokines, such as tumor growth factor-β (TGF-β)^39^. An experimental research of the gene expression profiles related to colorectal cancer shows that MEP1A is a prognostic biomarker and promotes proliferation and invasion of cancer^40^. The mechanism of MEP1A promoting cancer development may be realized by changing the expression of MMP9, vimentin, and E-cadherin, and participating in the EMT procession^41^. Another example indicates that DUSP26 associates with N-cadherin-mediated cell–cell adhesion, and downregulation of DUSP26 may contribute to malignant phenotypes of glioma^42^. Another study found that DUSP26 suppression intensively reduced the proliferation, EMT process and pEGFR expression in NSCLC cells. The specific mechanism is through facilitating ROS production and DNA damage and cell death. However, opposite phenotype was observed in NSCLC cells over-expressing DUSP26^43^.

Mutation of driving gene is closely related to the oncogenesis and progression of lung cancer. Particularly, NSCLC with early mutation can help us to identify a small proportion of LUAD population with risk of LNM as early as possible, which can greatly benefit from preventive adjuvant therapy. Genetic factors associated with an increased risk of LNM are not certain. One study involving in 675 patients with early LUAD showed that ALK rearrangement was more common in patients with LNM than EGFR mutation, while no obvious difference was observed between EGFR, KRAS and wild-type mutations in LNM^44^. Our study showed that the probability of EGFR, ALK and HER2 mutations in low-risk patients of the risk model is much higher, while the risk of ROS1 and BRAF mutations in high-risk patients is higher. Another study of patients with completely resected stage IA LUAD indicated that ALK rearrangement was correlated with more regional LNM and adverse disease-free survival compared with ALK negative groups^45^. In the prevalence analysis of ROS1 fusion, ROS1 status was significantly associated with LNM, and ROS1 positive rate was found to be high in patients with advanced lymph nodes stages^46^.

Currently, tumor stage has been broadly utilized as a strong indicator of survival in NSCLC^47,48^. However, the current staging system is far from accurate in the aspect of survival prediction at the individual level^49^. Combined with risk model and clinicopathological features, nomogram was developed to further improve the prediction ability and specificity of overall survival rate. In order to evaluate the accuracy of the predicted signal, we performed a time-dependent ROC analysis and calculated the AUC of different cut-off times. In the TCGA cohort, AUC obtained 0.806, 0.798 and 0818 at 3-, 5- and 7-years, respectively. There are other nomograms with good prognostic accuracy and clinical applicability in predicting lung cancer, but AUC is lower than ours^50,51^. As expected by our study, univariate and multivariate analysis showed that in TCGA database, N-stage, T-stage, tumor status and risk model were independent prognostic factors for OS. In the clinicopathological stratified survival analysis, patients with the same age, gender, stage, tumor status and recurrence status were divided into high, medium and low score subgroups. The results showed that the nomogram could still identify high-risk patients in the same subgroup. These results show that our 11 gene risk classifier and nomogram improve the prediction accuracy of survival in LUAD patients.

To the best of our knowledge, the 11-gene risk model had never been previously reported, and the nomogram that combined expressional information and clinicopathological factors would help clinicians to identify new prognostic biomarkers in LUAD from both a clinical and a basic perspective.

However, the limitations of our study should be recognized. First, this is a retrospective design study, and the sample size of the cohort is relatively small. Secondly, the expression and function of these 11 genes in patient tissues were not verified in the experiment. Further research is needed to reveal the interaction between these genes and verify our findings.

## Conclusion

Most importantly, our study revealed an 11-gene risk model based on TCGA LUAD cohort. The model was validated in groups with different disease characteristics. The model has high prediction accuracy for the overall survival rate of LUAD patients. In addition, we established and verified a LUAD prognostic nomogram composed of 11 gene risk model and clinicopathological features. The nomogram can also be used as a prediction tool for patients in different subgroups. Future experimental and clinical studies need to confirm our results.

## Supplementary Information

Supplementary Figure S1.

Supplementary Figure S2.

Supplementary Figure S3.

Supplementary Figure S4.

Supplementary Figure S5.

Supplementary Legends.

## Data Availability

The direct links required to find each data set in the database are as follows: the GEO gene expression and clinical pathology data set: https://ftp.ncbi.nlm.nih.gov/geo/series/GSE31nnn/GSE31210/matrix/ and https://ftp.ncbi.nlm.nih.gov/geo/series/GSE72nnn/GSE72094/matrix/; : The TCGA data underlying this study are freely available from The Cancer Genome Atlas (TCGA) via GDC data portal at https://portal.gdc.cancer.gov/projects/TCGA-LUAD. The data set downloaded by this direct link is the original data set. The authors did not have special access privileges.
