# Supplementary figures and images for: An eleven-gene risk model associated with lymph node metastasis predicts overall survival in lung adenocarcinoma
Source: Sci Rep. 2023 Apr 26;13:6852. doi: 10.1038/s41598-023-27544-0 (PMC10133305; doi:10.1038/s41598-023-27544-0)

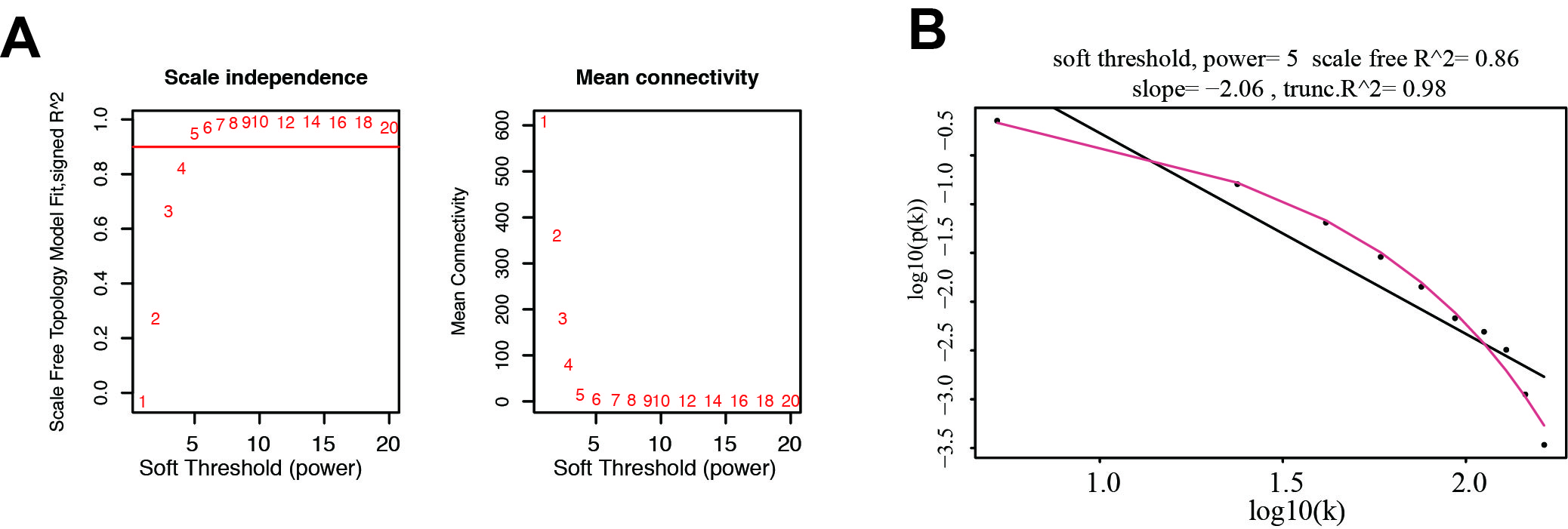

Supplement: Supplementary file 1 — Supplementary Figure S1. [file 41598_2023_27544_MOESM1_ESM.jpg]

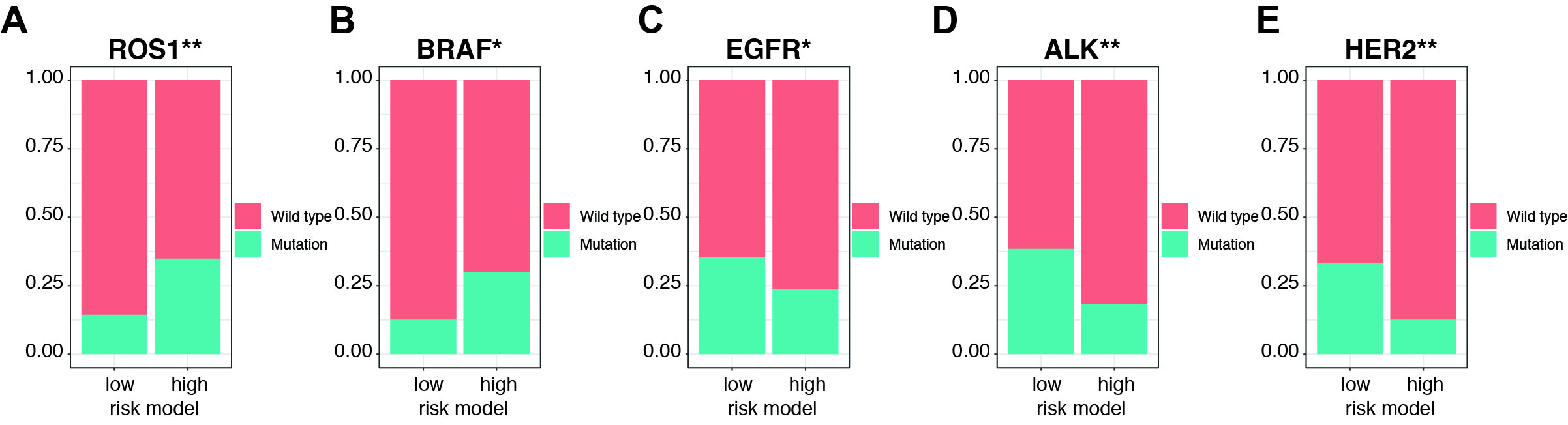

Supplement: Supplementary file 2 — Supplementary Figure S2. [file 41598_2023_27544_MOESM2_ESM.jpg]

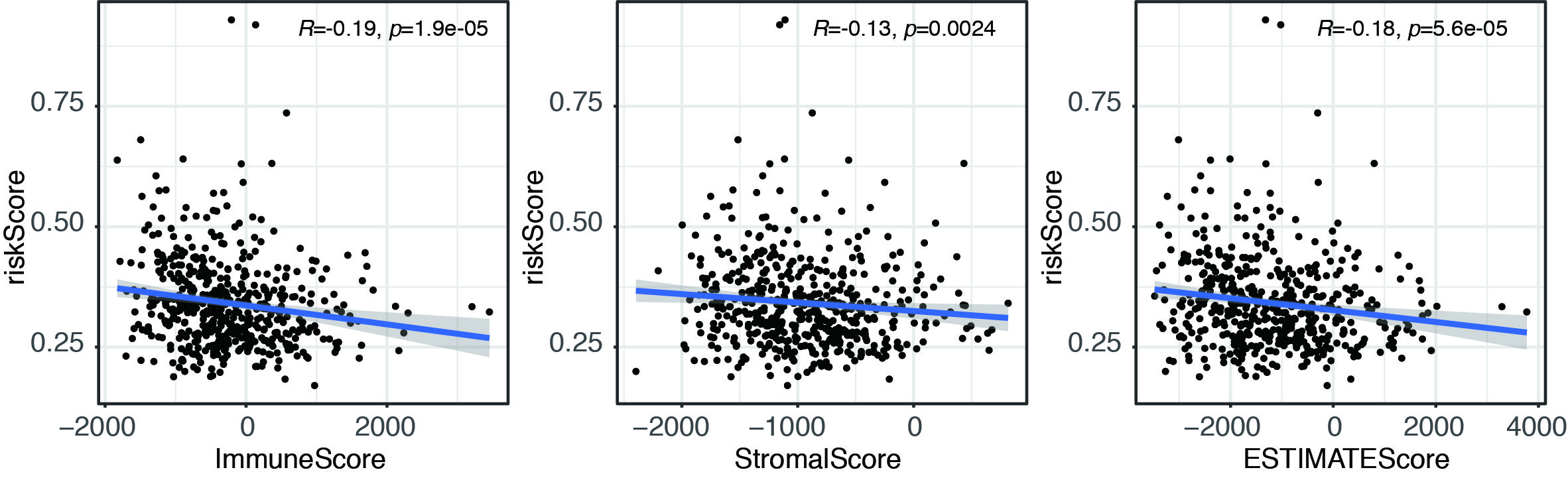

Supplement: Supplementary file 3 — Supplementary Figure S3. [file 41598_2023_27544_MOESM3_ESM.jpg]

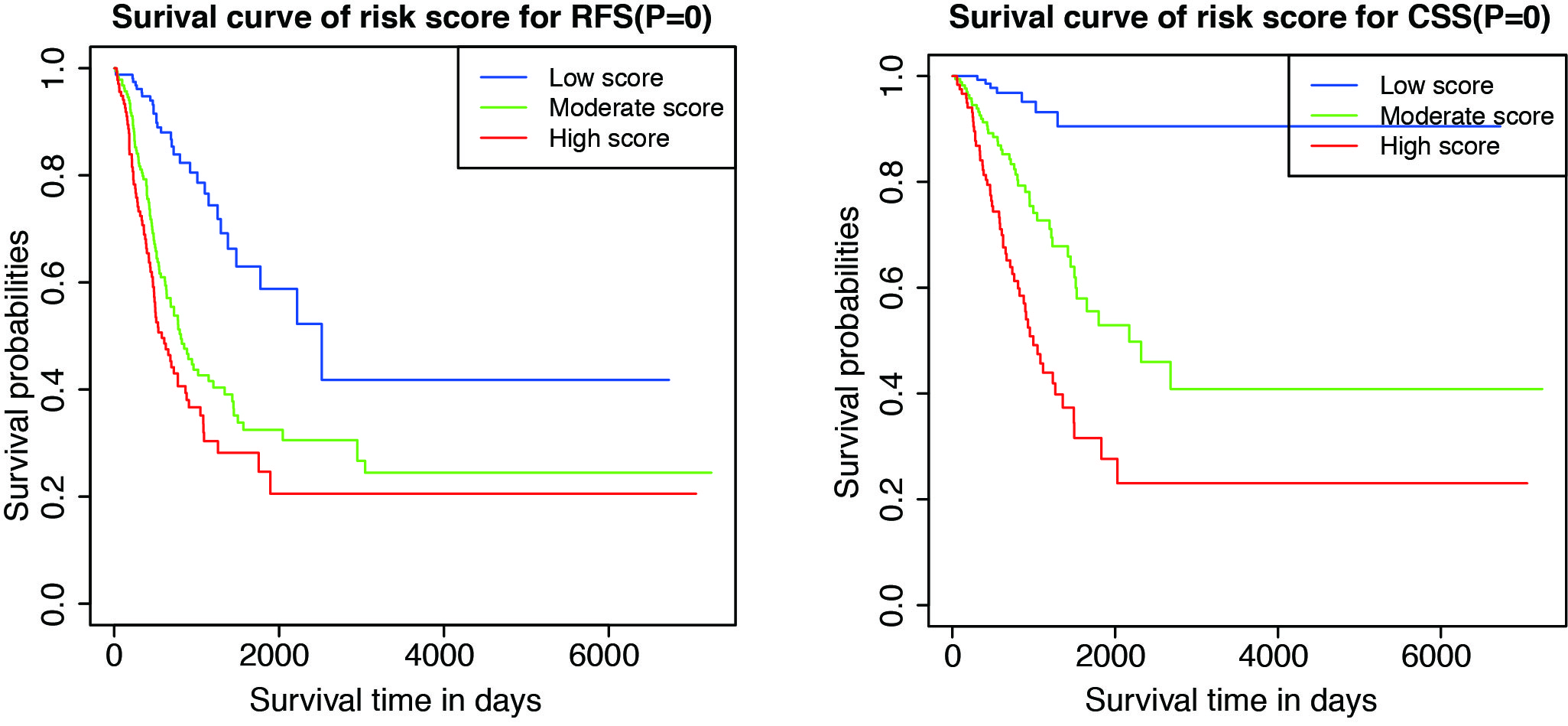

Supplement: Supplementary file 4 — Supplementary Figure S4. [file 41598_2023_27544_MOESM4_ESM.jpg]

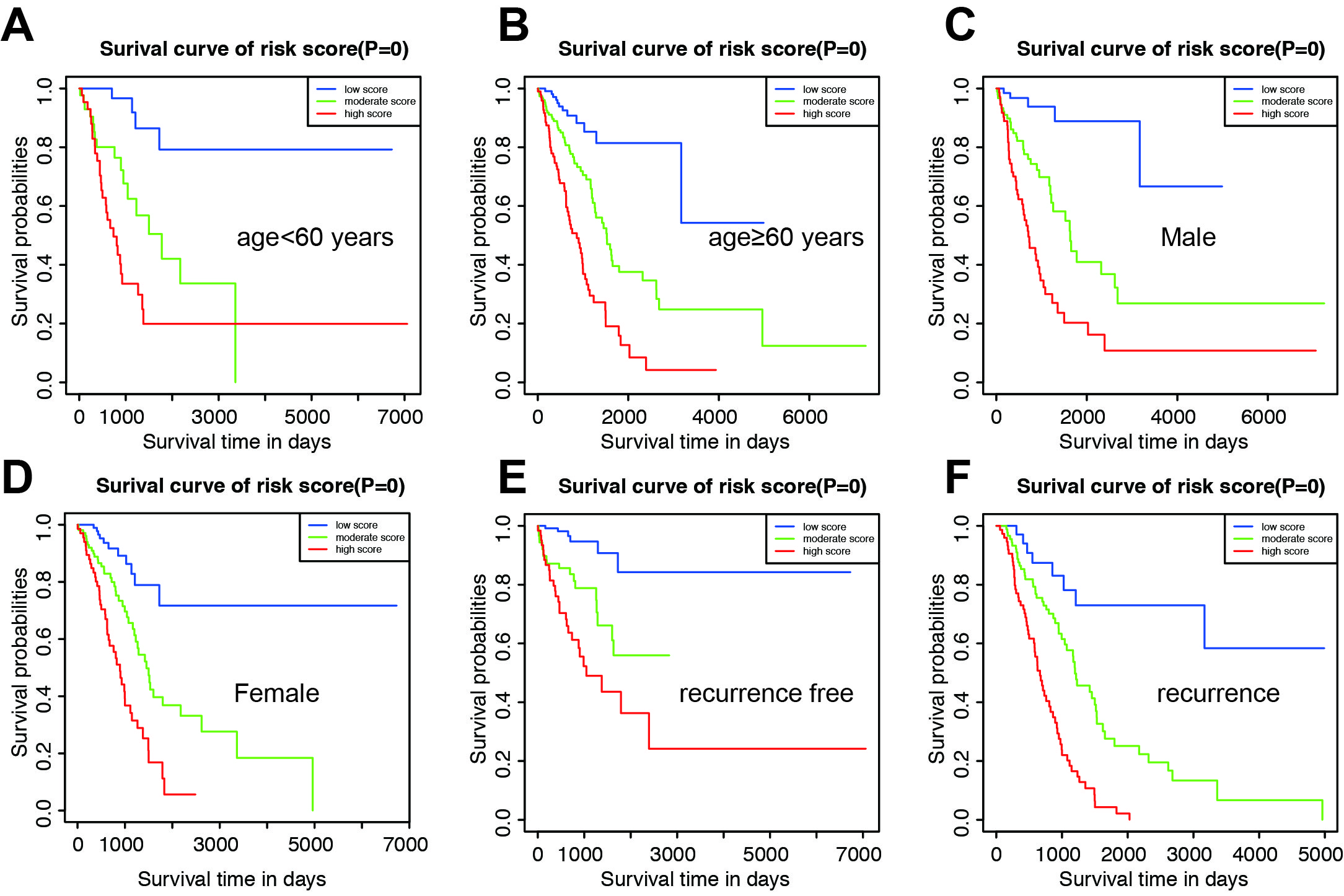

Supplement: Supplementary file 5 — Supplementary Figure S5. [file 41598_2023_27544_MOESM5_ESM.jpg]
