# Supplementary material for: An eleven-gene risk model associated with lymph node metastasis predicts overall survival in lung adenocarcinoma
Source: Sci Rep. 2023 Apr 26;13:6852. doi: 10.1038/s41598-023-27544-0 (PMC10133305; doi:10.1038/s41598-023-27544-0)
Supplement: Supplementary file 6 — Supplementary Legends. [file 41598_2023_27544_MOESM6_ESM.docx]

**Figure S1**: (A) Screening and (B) validation of the soft threshold.

**Figure S2**: Proportion of patients in different risk groups with different mutation genes. (A) ROS1. (B) BRAF. (C) EGFR. (D) ALK. (E) HER2. * *P* < 0.05, ** *P* < 0.01.

**Figure S3**: Correlation between risk score and immune score, stromal score, ESTIMATE score.

**Figure S4**: Survival curve of 11-gene risk model for RFS and CSS. RFS, recurrence-free survival; CSS, cancer-specific survival.

**Figure S5**: Prognostic value of the nomogram in different clinicopathological subgroups. (A) Age < 60 years old. (B) Age ≥ 60 years old. (C) Male. (D) Female. (E) Recurrence free group. (F) Recurrence group.
